# Impact of the COVID-19 pandemic on subcutaneous venous port-related complications in patients with cancer: a retrospective case–control study

**DOI:** 10.1186/s12957-022-02568-4

**Published:** 2022-03-31

**Authors:** Linnea Dahlin, Knut Taxbro, Fredrik Hammarskjöld

**Affiliations:** 1 Department of Anaesthesia and Intensive Care Medicine, Ryhov County Hospital, Jönköping, Sweden; 2 Faculty of Medicine and Health Sciences, Linköping University, Linköping, Sweden; 3 Department of Biomedical and Clinical Sciences, Linköping University, Linköping, Sweden

**Keywords:** Vascular access device, Central venous catheter thrombosis, COVID-19/SARS-COV-2, Complications, Neoplasms

## Abstract

**Background:**

Vascular access in cancer patients is of great importance in order to deliver tumour-specific therapy and continues to be so during exceptional conditions. This study aimed to examine the impact of the coronavirus disease 2019 pandemic on the care and complication rates associated with subcutaneous venous port (PORT) insertion in cancer treatment.

**Methods:**

We retrospectively studied all adult cancer patients that received a PORT in 2020 at a Swedish county hospital, including insertion characteristics and in-dwell complication rates for up to 6 months after implantation; these estimates were compared with historic data.

**Results:**

Data from 257 patients, of which 56 were haematological patients, were included and compared with those of 168 patients in the control group. The group characteristics were similar, except for the inclusion of haematological patients in the study group. Insertion characteristics showed a shorter waiting time and higher rates of antibiotic and sedative use during the pandemic. The rates of postoperative haematoma and catheter occlusion during the study period were higher than otherwise. The rates of adverse events related to the PORT in the solid tumour group were comparable to those in the control group (18.4% vs. 14.9%). Patients with haematological malignancies were more likely to experience adverse events (37.5% vs. 18.4%) and deep venous thrombosis (7.1% vs. 1.0%) than those with solid tumours.

**Conclusion:**

In conclusion, the present findings suggest that PORTs remain a safe venous access system even during a pandemic, indicating a robust vascular access service.

## Introduction

In modern oncologic care, access to the central venous system for the delivery of tumour-specific therapy is paramount. Long-term central venous catheters (CVC), including subcutaneous venous ports (PORTs), are frequently used to provide safe venous access during treatment [1]. Like other CVC types, PORTs are associated with complications, which may affect treatment and, in some cases, be life-threatening [2, 3]; however, the risk of complications may be reduced when working according to evidence-based strategies [4, 5].

The outbreak of coronavirus disease 2019 (COVID-19) occurred in the winter of 2019 [6] and has since caused a disruption to healthcare systems. The first case of COVID-19 in Sweden was detected at our hospital on 31 January 2020, in a region that became one of the areas most affected by the pandemic [7]. A substantial part of all physicians, nurses, and nurse assistants in the operating theatre were relocated to work in the COVID intensive care unit. Hence, the number of operations was greatly reduced, and extensive priorities between surgical cases were made. This development has put the local Department of Anaesthesia and Intensive Care Medicine under substantial stress. This department is responsible for the insertion of PORTs, a service it continued to provide during the pandemic. Meanwhile, departments of both oncology and haematology were involved in treating COVID-19 patients. These factors may have negatively affected PORT insertion rates and the associated quality of care, increasing the rates of complications. Studies on the availability of vascular access services for patients with cancer during a pandemic remain limited. The present study aimed to examine whether the rates of PORT availability, care, and insertion-related complications during the pandemic were comparable with those previously reported by our hospital, as part of the PICCPORT trial [8]. We hypothesised that these factors could be affected by extensive system reorganisation and staff shortages.

## Methods

### Setting

The data were collected at the Ryhov County Hospital in Jönköping, Sweden, with a catchment population of 360,000 inhabitants. Approximately 300 PORTs are inserted annually at the hospital, and the main indication is cancer treatment.

### Patients

The study group included cancer patients (age ≥ 18 years) who received a PORT between 1 January 2020 and 31 December 2020 with the intention of receiving intravenous chemotherapeutic treatment during the study period. The control group were patients with cancer included in the PICCPORT trial from Jönköping between March 2013 and February 2017 [8]. The patients were included in the PICCPORT trial if the responsible oncologist considered the patient suitable for both peripheral inserted central catheter (PICC) or PORT and the patient accepted randomisation. The trial assessed 1597 patients for eligibility, and patients 18 years old with a life expectancy longer than 4 weeks and requiring chemotherapy through a CVC were eligible for inclusion. Ongoing severe systemic infection, clinically significant upper extremity/central DVT, severe coagulopathy, inability to communicate, or an imminent need for a dialysis fistula were exclusion criteria.

### Data collection

Patients were identified using a patient data management system (Metavision^TM^ version 5.46, iMDsoft, Israel). Data were obtained from this system and from an electronic patient data file (Cosmic^TM^ version R8.2.07, Cambio Healthcare Systems AB, Sweden).

Data on the following variables were collected at the insertion date: patient characteristics (age, sex, cancer diagnosis, intention of cancer treatment), implantation characteristics (operator specialty [anaesthesiologist or surgeon], type of anaesthesia [local with or without sedation], preoperative antibiotics used, choice of vessel, preoperative fluoroscopy, real-time ultrasound, and procedure time), and complication rates (pneumothorax, bleeding, and arterial puncture).

During the follow-up period after PORT insertion, the following complications were assessed: infection (local, catheter-related infection, and catheter-associated bloodstream infection [CRBSI]), causative microorganisms, thrombosis, catheter occlusion, catheter fracture, rotation of the chamber, and catheter dislodgment.

All data were retrospectively collected according to the study protocol used in the PICCPORT trial [8]. All inserted PORTs during the year 2020 were included in the study. If the PORT was removed prior to minimum follow-up due to complication, death, or end of treatment, the patient was not further followed. The median follow-up time was 180 (IQR 66) days. The 49 patients with a PORT inserted after October 1 who did not have their port removed or died were followed up for a median of 134 (IQR 42) days for practical reasons.

The patients in the PICCPORT trial were followed up for up to 12 months. To increase accuracy, all infectious and thrombus complications were evaluated by two of the authors (LD and FH).

### PORT care and outcome definitions

During 2019, the protocol for inserting a needle into the PORT was updated due to an increase in the incidence of local infections; consequently, sterile draping was mandatory during PORT chamber cannulation.

Antibiotics during insertion included both single-dose antibiotics, and ongoing treatment for infections.

Haematomas were defined as requiring intervention, including, at a minimum, a change or strengthening of the dressing over the surgical wound.

Catheter-related deep venous thrombosis (DVT) was defined as symptoms or clinical signs of DVT (pain, redness, swelling, and tenderness in a relevant area) and confirmed by ultrasound/computed tomography scanning or a DVT incidentally found on imaging examination performed for other purposes [2].

Catheter occlusion was defined as the inability to aspirate or flush via the catheter, where alteplase or ethanol instillation was required to resolve the occlusion.

Catheter-associated infections were defined according to the Infectious Diseases Society of America criteria:

Catheter colonisation: Significant growth (> 1 colony-forming unit (CFU)) in a culture from the pocket, catheter tip, or segment.

Local infection:

- Microbial definition: positive culture and exudate from the exit site
- Clinical definition: inflammation in an area < 2 cm over the PORT

CRI: Positive culture from the tip of the catheter systemic inflammatory symptoms, there is no other obvious source of the infection.

CRBSI: Positive culture from peripheral drawn blood and symptoms of a systemic inflammation. There should be no other obvious source of infection. In addition, at least one of the following present:

- Indistinguishable microorganisms found on tip and blood culture
- Paired positive blood culture [9]

Mechanical complications were defined as events related to the placement of a PORT and its usage. For example, a catheter tip was placed inside the right ventricle, the infusion chamber had rotated, or any type of damage to the system that interfered with its usage was observed. All grade adverse events were created as a composite variable containing DVT, occlusion, catheter-associated infection, and mechanical complications.

### Statistical analysis

Descriptive analyses were performed to characterise the patient population. Pearson’s *χ*^2^, Fisher’s exact, Mann–Whitney *U*, and Cox regression tests were used to compare groups depending on whether the data were discrete or continuous and whether the distribution of data was normal or non-normal. A multivariable Cox proportional hazards model was used to identify independent predictors (treatment goal, sex, age, cancer type, vessel, operator, and PORT insertion timing) of catheter-related DVT and catheter-related adverse events (thrombosis, occlusion, infection, mechanical issues, or death) during the follow-up period. Predictors with *p*-values of > 0.05 were manually removed from the model. Analyses of complication-free catheter survival were performed using the Kaplan–Meier method and log-rank test. Due to the difference in follow-up time in the study group, a sensitivity analysis was performed where the patients with a shorter follow-up time were excluded, and overall complications were compared with the control group. All analyses were conducted using a statistical software package (SPSS version 27.0, Armonk, NY, USA).

## Results

**Fig. 1:**
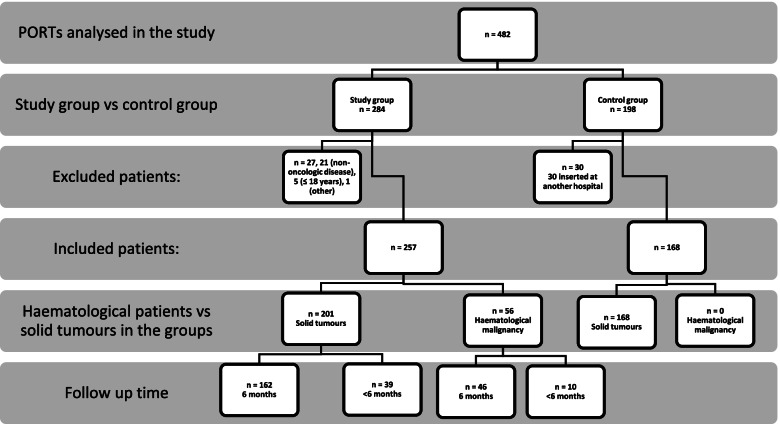
Study flow diagram

A total of 284 PORTs were inserted in 2020, of which 257 met the study inclusion criteria. Twenty-seven patients were excluded from this study (Fig. 1). The control group included 198 PORTs, of which 30 were excluded (Fig. 1). The patients’ baseline characteristics are shown in Table 1. Age, sex, and treatment strategy did not differ between the groups, but there were significant differences in cancer diagnoses (*p* < 0.001). Forty-three (16.7%) and 34 (20.2%) patients in the study and control groups died during the follow-up period, respectively; none of the deaths was related to PORT complications.

**Table 1:** Baseline characteristics of all patients in the study and control groups

	Study group	Control group	*p*-value
	*n* = 257	*n* = 168	
Sex *n* (%)			0.901
Female	153 (59.5)	99 (58.9)	
Male	104 (40.5)	69(41.1)	
Age (years) median (min–max)	65 (19–89)	65 (30–89)	0.864
Cancer, *n* (%)			< 0.001
Breast	60 (23.3)	67 (39.9)	
Colorectal	39 (15.2)	35 (20.8)	
Upper GI	40 (15.6)	20 (11.9)	
Urogenital	13 (5.1)	27 (16.1)	
Haematologic cancer	56 (21.8)	0 (0)	
Others	26 (10.1)	19 (11.3)	
Gyn	23 (8.9)	0 (0)	
Treatment strategy, *n* (%)			0.182
Adjuvant	147 (57.2)	107 (63.7)	
Palliative	110 (42.8)	61 (36.3)	

*GI* gastrointestinal, *Gyn* gynaecological

The insertion data are presented in Table 2, revealing an increased use of sedation (64 [24.9%] vs. 15 [8.9%], *p* < 0.001) and median procedural time (33 min vs. 26 min, *p* < 0.001) in the study group compared to those in the control group. In the solid tumour group, preoperative antibiotics were used in 22/201 (10.1%) patients compared to 2/168 (1.2%) patients in the control group (*p* < 0.001). Preoperative antibiotics were used in 26/56 (46.1%) haematological patients.

**Table 2:** PORT insertion characteristics and related complications in the study and control group patients

		Study group	Control group	*p*-value
		*n* = 257	*n* = 168	
Waiting time (days) median (IQR)		8 (7)	12 (7)	< 0.001
Inserting clinician, *n* (%)				0.004
	Anaesthetist	245 (95.3)	168 (100)	
	Surgeon	12 (4.7)	0 (0)	
Vein, *n* (%)				0.193
	Internal jugular	251 (97.7)	160 (95.2)	
	Subclavian	3 (1.2)	4 (2.4)	
	Femoral	3 (1.2)	0 (0)	
	Unknown	0 (0)	4 (2.4)	
Side, *n* (%)				0.343
	Right	217 (84.4)	131 (78.0)	
	Left	40 (15.6)	31 (18.5)	
	Unknown	0 (0)	6 (3.6)	
Anaesthetic, *n* (%)	LA	192 (74.7)	149 (88.7)	
	LA + sedation	64 (24.9)	15 (8.9)	< 0.001
	General anaesthetic	1 (0.4)	1 (0.6)	–
	Unknown	0 (0)	3 (1.8)	–
Ultrasound, *n* (%)				0.387
	Yes	253 (98.4)	163 (97.0)	
	No	2 (0.8)	3 (1.8)	
	Unknown	2 (0.8)	2 (1.2)	
Fluoroscopy, *n* (%)				0.059
	Yes	253 (98.4)	152 (90.5)	
	No	2 (0.8)	6 (3.6)	
	Unknown	2 (0.8)	10 (6.0)	
Arterial puncture, *n* (%)	Yes	1 (0.4)	1 (0.6)	1.0
Pneumothorax, *n* (%)	Yes	0 (0)	0 (0)	–
Haemothorax, *n* (%)	Yes	0 (0)	0 (0)	–
Haematoma, intervention required, *n* (%)	Yes	13 (5.1)	2 (1.2)	0.036
Antibiotics during insertion, *n* (%)	Yes	48 (18.7)	2 (1.2)	< 0.001
Procedure time, median (IQR)		33 (13)	26 (11)	< 0.001

*IQR* interquartile range, *LA* local anaesthetic

The median waiting time for receiving a PORT was shorter during than before the pandemic (8 days vs. 12 days, *p* < 0.001). The only difference in insertion complications between the two groups was an increase in the number of postoperative haematomas in the study group (13 [5.1%] vs. 2 [1.2%], *p* = 0.036). Twelve of the haematomas were managed with dressing strengthening, and one required a surgical revision.

The median catheter dwell time per patient was shorter in the solid tumour group than in the control group (175 days vs. 217 days, *p* < 0.001).

**Fig. 2:**
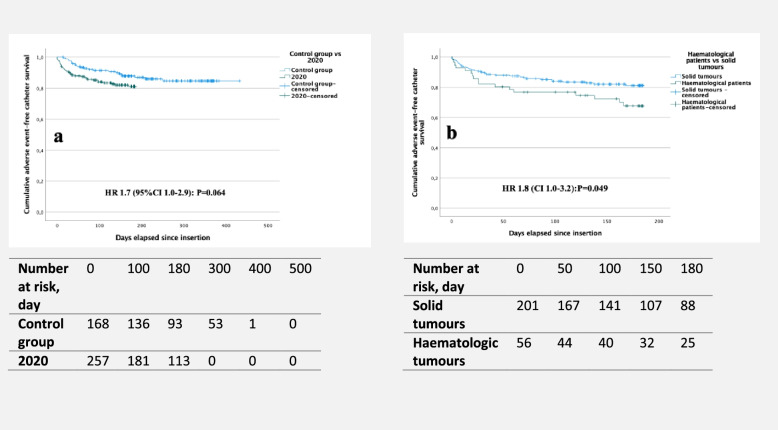
**A** Comparisons of cumulative adverse event-free catheter survival rates between solid tumour patients with a PORT in the study and control group**s**. **B** Comparisons of cumulative adverse event-free catheter survival rates between patients with haematological malignancies and solid tumours in the study group. *p*-values were derived with the log-rank test. CI, confidence interval. HR, hazard ratio

In-dwell characteristics and complications in patients with solid tumours in the study and control groups are presented in Table 3. There was no significant difference in all-grade adverse events (37 vs. 25, *p* = 0.068) as presented in Fig. 2A. However, the median time to the first event was 22 days in patients with solid tumours, compared to 60 days in the control group (*p* < 0.001), and the incidence of occlusion was higher in the study group than in the control group (18 cases vs. 1 case, *p* = 0.007).

**Table 3:** In-dwell characteristics and adverse events of PORT-inserted patients, stratified by solid tumours versus the control group

Complications, haematological cancer — solid tumours	2020 solid tumours	Control group	HR	95% CI	*p*-value
	*n* = 201	*n* = 168			
Total catheter days	29,071	37,469	–	–	–
CD per patient, median (IQR)	175 (69)	217 (179)	–	–	< 0.001
Mortality, *n* (%)	36 (17.9)	34 (20.2)	1.5	0.9–2.6	0.121
DVT, *n* (%)	2 (1.0)	1 (0.6)	2.0	0.2–21.9	0.578
DVT/1000 CD	0.07	0.03	–	–	–
Days to DVT, median (IQR)	85 (−)	–	–	–	–
All catheter infections, *n* (%)	9 (4.5)	16 (9.5)	0.6	0.3–1.4	0.211
*Infection/1000 CD*	0.31	0.43	–	–	–
*Local infection, n (%)*	9 (4.5)	15 (8.9)	–	–	–
*CRI, n (%)*	3 (1.5)	–	–	–	–
*CRBSI, n (%)*	2 (1.0)	2 (1.2)	1.8	0.2–20.1	0.623
*CRBSI/1000 CD*	0.07	0.05	–	–	–
Days to infection median (IQR)	56 (85)	46 (96)	–	–	0.428
Haematoma, *n* (%)	10 (5.0)	2 (1.2)	–	–	0.043
Antibiotics, *n* (%)	22 (10.1)	2 (1.2)			< 0.001
Mechanical, *n* (%)	6 (3.0)	7 (4.2)	1.0	0.3–3.0	0.945
Mechanical/1000 CD	0.21	0.19	–	–	–
Days to mechanical event median (IQR)	16 (57)	122 (107)	–	–	0.015
Occlusion, *n* (%)	18 (9.0)	1 (0.6)	16.2	2.2–121.1	0.007
Occlusion/1000 CD	0.62	0.03			–
Days to occlusion median (IQR)	17 (43)	– (−)	–	–	–
All grade adverse events, *n* (%)	37 (18.4)	25 (14.9)	1.7	1.0–2.9	0.068
All grade adverse events/1000 CD	1.27	0.67	–	–	–
Days to all grade adverse events median (IQR)	22 (63)	60 (114)	–	–	0.001

*HR* hazard ratio, *CI* confidence interval, *CD* catheter days, *IQR* interquartile range, *DVT* deep venous thrombosis, *CRI* catheter-related infection, *CRBSI* catheter-related bloodstream infection

A comparison of haematological and solid tumours during the study period is shown in Table 4. The median number of catheter days per patient in the haematological and solid tumour groups was 181 and 175, respectively (*p* = 0.496). The incidence of DVT was significantly higher in the haematological group than in the solid tumour group (4 [7.1%] vs. 2 [1.0%], *p* = 0.021).

**Table 4:** In-dwell characteristics and adverse events of PORT-inserted patients, stratified by solid tumours versus haematological malignancies

	Haematological malignancy	Solid tumours	HR	95% CI	*p*-value
	*n* = 56	*n* = 201			
Total catheter days	8226	29,071	–	–	–
CD per patient, median (IQR)	181 (63)	175 (69)	–	–	0.496
Mortality, *n* (%)	7 (12.5)	36 (17.9)	0.7	0.3–1.6	0.416
Days to death median (IQR)	100 (111)	67 (76)	–	–	0.1
DVT, *n* (%)	4 (7.1)	2 (1.0)	7.3	1.3–40.1	0.021
DVT/1000 CD	0.49	0.07	–	–	–
Days to DVT median (IQR)	15(36)	85 (−)	–	–	–
All catheter infections, *n* (%)	4 (7.1)	9 (4.5)	1.6	0.5–5.3	0.42
Infection/1000 CD	0.49	0.31	–	–	–
*Local infection, n (%)*	3 (5.4)	9 (4.5)	1.2	0.3–4.5	0.766
*CRI, n (%)*	3 (5.4)	3 (1.5)	–	–	0.12
*CRBSI, n (%)*	3 (5.4)	2 (1.0)	5.5	0.9–33.1	0.061
*CRBSI/1000 CD*	0.36	0.07	–	–	–
Days to infection median (IQR)	23 (37)	56 (85)	–	–	0.643
Haematoma, *n* (%)	3 (5.4)	10 (5.0)	–	–	1.0
Antibiotics, *n* (%)	26 (46.4)	22 (10.1)	–	–	< 0.001
Mechanical, *n* (%)	3 (5.4)	6 (3.0)	1.8	0.5–7.3	0.398
Mechanical/1000 CD	0.36	0.21	–	–	–
Days to mechanical event, median (IQR)	4 (−)	16 (57)	–	–	0.604
Occlusion, *n* (%)	8 (14.3)	18 (9.0)	1.6	0.7–3.7	0.256
Occlusion/1000 CD	0.97	0.62	–	–	–
Days to occlusion, median (IQR)	39 (144)	17 (43)	–	–	0.317
All grade adverse events, *n* (%)	21 (37.5)	37 (18.4)	1.8	1–3.2	0.053
All grade adverse events/1000 CD	2.55	1.27	–	–	–
Days to all grade adverse events, median (IQR)	26 (82)	22 (63)	–	–	0.64

*HR* hazard ratio, *CI* confidence interval, *CD* catheter days, *IQR* interquartile range, *DVT* deep venous thrombosis, *CRI* catheter-related infection, *CRBSI* catheter-related bloodstream infection

The Kaplan–Meier curve presented in Fig. 2B shows a significantly higher risk (21 [37.5%] vs. 37 [18.4%], *p* = 0.049) of all adverse events related to PORTs in haematological patients than in solid tumour patients.

Infections related to PORTs in the study group were caused by coagulase-negative staphylococci in seven cases, *Staphylococcus aureus* in five cases, and mixed bacterial growth in one case.

Nineteen (7.4%) and 19 (11.3%) PORTs in the study and control groups were removed prematurely due to complications, respectively. Reasons for PORT removal in the study group were infection (52.6%), mechanical complications (31.6%), and DVT (one case, 5.3%); in the control group, these reasons were infection (68.4%) and mechanical events (31.5%). When excluding the PORTs followed for less than 6 months, 162 patients with solid tumours were compared with the 168 patients in the control group. There was no significant difference in overall complications between the solid tumour patients followed for 6 months compared with the control group (31 [19.1%] vs. 25 [14.9%], *p* = 0.135).

## Discussion

Even during a healthcare crisis, reliable and safe venous access for patients with cancer is key to good outcomes. The present findings suggest that cancer patients were able to obtain PORTs for cancer treatment within a short time and with low complication rates, which were similar to those observed pre-pandemic [2, 8].

There are limited data on complications related to venous access during the pandemic. One recently published study analysed the effect of prolonged PORT flushing intervals during the pandemic and found no difference in occlusion rates between groups with different duration intervals [10]. However, studies that examined CRBSI incidence during the pandemic found an increase in the rates of this complication, likely due to a decrease in preventive hygiene protocols [11, 12]. Meanwhile, an observational study reported a lower incidence of infection related to haemodialysis catheters due to the implementation of updated hygiene protocols during the pandemic [13]. In contrast, other clinical situations, such as ST elevation myocardial infarction, have shown increased morbidity and mortality during the pandemic [14]. Another study reported that the incidence of short-term complications related to appendicitis remained unchanged [15]. Oncology patients often have special prerequisites for their treatment which make them vulnerable during the COVID-19 pandemic. An increased waiting time can affect their prognosis negatively and thorough routines should be instituted to prevent hospital transmission of COVID-19 to minimise the risk of infection [16, 17].

Some studies have shown a decrease in the incidence of oncology diagnosis and oncology surgery. This is probably due to patients not seeking health care in fear of being infected with COVID-19, and additionally, oncology strategies have changed to less surgery due to decreased operating capability [16, 18]. Overall, this evidence suggests that the COVID-19 pandemic may have had a different impact on outcomes in different healthcare system areas.

Nevertheless, the waiting time for PORT implantation was shorter during than before the pandemic. This finding may be explained by the explicit strategy for maintaining normal standards for cancer treatment during this time. In addition, the results from the PICCPORT trial, which favoured PORTs over peripherally inserted central catheters (PICCs), may have influenced the efforts to increase the availability of PORT insertion. The number of inserted PORTs during the pandemic was approximately the same as that recorded in the previous years; hence, fewer inserted PORTs may not explain the decreased waiting time. However, we have not analysed if there is a change in treatment strategies from surgery to other treatments during the pandemic.

We believe that strict adherence to well-established evidence-based protocols (ultrasound-guided puncture, use of fluoroscopy, and maximal sterile precautions [19]) may explain the low rate of insertion-related complications. However, the incidence of uncomplicated postoperative haematomas was relatively high, among both haematological patients and in the solid tumour group. The reasons for this increase remain unclear and may be related to the involvement of less experienced operators or some patient factors (i.e. use of anticoagulants or thrombocyte dysfunction), as the insertion technique remained unchanged; future studies are required to elucidate this phenomenon, as postoperative bleeding may increase the risk of early infections [20].

The rate of procedural sedation use increased during the pandemic. In the control group, a high pain level during insertion was reported in 25% of cases, regardless of sedation [8]. To minimise pain and patient discomfort during insertion, clinicians may have adapted a more liberal approach to sedation. The Swedish CVC guidelines do not recommend a type of anaesthetic to be used during insertion [3]. Studies performed on patients’ satisfaction reported overall high satisfaction when using only a local anaesthetic [21], and rates of discomfort do not automatically differ between patients receiving a local anaesthetic combined with sedation and those receiving only a local anaesthetic [3]. Therefore, the use of sedation during PORT insertion should be evaluated in randomised controlled trials (RCTs).

Preoperative antibiotic prophylaxis during PORT insertion is controversial [3]; however, its use has increased during the pandemic. This increase is only partially explained by the inclusion of patients with haematological diseases, uniquely susceptible to infection. The indications for prophylactic antibiotics in hospitalised patients include neutropenia or complicated insertion [3], suggesting that antibiotic use may be influenced by the subjective evaluation of a complicated insertion made by the operator. The preoperative use of antibiotics should be further examined in RCTs.

Occlusion of the catheter may delay cancer treatment and, occasionally, catheter replacement, increasing the risk of complications. The occlusion rate in the control group was very low, and one patient experienced occlusion that required thrombolysis to restore lumen patency. Since the control group was part of an RCT comparing PORTs and PICCs, a great focus was placed on central venous access protocols. This focus may have resulted in very good adherence to protocols, and the Hawthorne effect may have contributed to the low occlusion rate in this study. The occlusion rate observed in this study was 9%, which is consistent with the corresponding 15% reported by a previous study [2]. In addition, we believe that the use of small-calibre (i.e. more susceptible to occlusion) PORT catheters has increased since the onset of the pandemic.

The incidence of catheter infection in the solid tumour group was 4.5% (0.31/1000 CD), which is consistent with that observed in our previous study [2, 8]. Other studies conducted before the pandemic reported an incidence of infection in the range of 4.0–18.0% [22, 23]. Our research group has shown in several studies that the incidence of catheter-associated infections is low [3, 8, 24, 25]. These findings may reflect the effects of well-established routines throughout the hospital, preventing the pandemic from negatively impacting infection prevention in patients receiving intravascular catheters. In addition, the pandemic has highlighted the importance of hand hygiene and other infection prevention strategies, which were put in place to limit the in-hospital transmission of COVID-19. The incidence of local PORT infection was high in the control group [8]. For this reason, the use of a sterile drape and gloves became mandatory in 2019 when cannulating the PORT chamber. This approach may have decreased the incidence of infection. Even though the infection rate was low (CRBSI, 0.07/1000 CD), it remained a major reason for premature device removal [26], which was also observed in this study. Nineteen PORTs were prematurely removed due to complications, of which 10 (52.6%) had a PORT-associated infection.

Haematological disease is a risk factor for overall complications (hazard ratio of 8.3 CI [1.1–64]). High-grade evidence-based recommendations on vascular access devices for haematological patients are limited because there are few RCTs with a haematology focus. In the present study, there was a tendency toward a higher incidence of separate complications in haematology patients than in solid tumour patients; however, only DVT rates were significantly different between the groups. Haematological patients may be at a higher risk of developing DVT [27]; however, the development of DVT in individual patients is multifactorial. We did not screen patients for other known DVT risk factors (obesity, previous DVT events, coagulation defects, or thrombogenic treatments), which could contribute to the difference between the groups [28].

The occlusion rate was not significantly higher in haematology patients than in solid tumour patients; however, the incidence remained high, with 8 (14.3%) patients affected. Haematological patients frequently receive blood products through PORT, thereby increasing the risk of intraluminal thrombotic formation [29]. In addition, haematologic and solid cancer patients are treated at separate departments, where adherence to protocols may have differed.

This study had several strengths. First, the control group data were acquired from a recently published RCT [8]. Second, the study patients were evaluated using the same protocol. Third, no patient was lost to follow-up. This study has several limitations that should be considered when interpreting its findings. First, the median number of catheter days per patient was lower in the study than in the control dataset since some of the PORTs in the study group were followed for shorter than 6 months. This leaves the possibility that some complications occurred after the end of data collection. However, most complications in the control group occurred before day 90, suggesting that most complications were included in the study. In addition, when excluding the patients followed for a shorter period than 6 months, no difference is observed in the incidence of overall complications. Second, due to the retrospective single-centre study design, the present findings may have limited external validity. Third, several characteristics of the pandemic were not accounted for, such as its impact on the incidence of cancer diagnosis, patients sent to other hospitals for treatment, changes in treatments to avoid the need for PORT insertion, and increasing numbers of inserted PICCs. Fourth, the haematologic study population was small, which limited data interpretation. Fifth, data were collected through a review of medical records and were susceptible to reporting bias.

In conclusion, this study has shown that insertion of PORTs for cancer treatment has been a prioritised surgical procedure during the COVID-19 pandemic. Furthermore, PORTs remain a safe venous access system even in exceptional pandemic conditions, indicating a robust vascular access service.

## Data Availability

The data that support the findings of this study are available from the corresponding author upon reasonable request.
